# Genomic complexity of urothelial bladder cancer revealed in urinary cfDNA

**DOI:** 10.1038/ejhg.2015.281

**Published:** 2016-01-13

**Authors:** Fiona S Togneri, Douglas G Ward, Joseph M Foster, Adam J Devall, Paula Wojtowicz, Sofia Alyas, Fabiana Ramos Vasques, Assa Oumie, Nicholas D James, K K Cheng, Maurice P Zeegers, Nayneeta Deshmukh, Brendan O'Sullivan, Philippe Taniere, Karen G Spink, Dominic J McMullan, Mike Griffiths, Richard T Bryan

**Affiliations:** 1 West Midland Regional Genetics Laboratory, Birmingham Women's NHS Foundation Trust, Birmingham, UK; 2 Institute of Cancer & Genomic Sciences, College of Medical and Dental Sciences, University of Birmingham, Birmingham, UK; 3 Affymetrix UK Ltd, High Wycombe, UK; 4 Cancer Research Unit, University of Warwick, Coventry, UK; 5 School of Health and Population Sciences, University of Birmingham, Birmingham, UK; 6 Department of Complex Genetics, NUTRIM School of Nutrition and Translational Research in Metabolism, Maastricht University Medical Centre, The Netherlands; 7 Department of Histopathology, University Hospitals Birmingham NHS Foundation Trust, Birmingham, UK

## Abstract

Urothelial bladder cancers (UBCs) have heterogeneous clinical characteristics that are mirrored in their diverse genomic profiles. Genomic profiling of UBCs has the potential to benefit routine clinical practice by providing prognostic utility above and beyond conventional clinicopathological factors, and allowing for prediction and surveillance of treatment responses. Urinary DNAs representative of the tumour genome provide a promising resource as a liquid biopsy for non-invasive genomic profiling of UBCs. We compared the genomic profiles of urinary cellular DNA and cell-free DNA (cfDNA) from the urine with matched diagnostic formalin-fixed paraffin-embedded tumour DNAs for 23 well-characterised UBC patients. Our data show urinary DNAs to be highly representative of patient tumours, allowing for detection of recurrent clinically actionable genomic aberrations. Furthermore, a greater aberrant load (indicative of tumour genome) was observed in cfDNA over cellular DNA (*P*<0.001), resulting in a higher analytical sensitivity for detection of clinically actionable genomic aberrations (*P*<0.04) when using cfDNA. Thus, cfDNA extracted from the urine of UBC patients has a higher tumour genome burden and allows greater detection of key genomic biomarkers (90%) than cellular DNA from urine (61%) and provides a promising resource for robust whole-genome tumour profiling of UBC with potential to influence clinical decisions without invasive patient interventions.

## Introduction

Urothelial bladder cancer (UBC) is the seventh most common cancer in Western societies with a rising global incidence.^1^ Disease management poses numerous challenges because of the following: (i) the propensity for non-muscle-invasive bladder cancer (NMIBC) to recur, necessitating long-term surveillance; (ii) a variable risk of NMIBC progression, associated with poor 5-year survival;^2, 3^ (iii) a lack of proven biomarker prognosticators to identify those subsets of patients who will suffer tumour recurrence, progression and death; and (iv) the radical therapies required to treat muscle-invasive disease (MIBC).^4^ UBCs are thus highly heterogeneous in their clinical characteristics and this is mirrored in their genomics, characteristics of which traverse conventional grade and stage groupings.^5^

Typically, genomic aberrations in tumours have been characterised using formalin-fixed paraffin-embedded (FFPE) or fresh-frozen tumour tissue, with such analyses elucidating promising biomarkers and suggesting genomic signatures with potential to influence future therapeutic interventions.^6, 7, 8^ Identifying such genomic complexity in a non-invasive manner could be highly advantageous for facilitating the diagnosis, treatment and surveillance of patients with NMIBC or MIBC.^9, 10^

Genetic changes in UBCs have previously been investigated non-invasively using genetic material present in the urine. Both genetic material from exfoliated cells (which pellet upon centrifugation) and cell-free DNA (cfDNA; which remains in the supernatant following centrifugation) have been studied. Most studies to date have focused on exfoliated cells, with data giving a specific read out, for example, the presence or absence of UBC.^11^ Urine tests looking at genomic copy number (CN) include the FISH-based UroVysion test (Abbott, Des Plaines, IL, USA; FDA-approved UBC diagnosis),^12^ which uses individual exfoliated tumour cells isolated from urine, and the CGH-based BCA-1 test, which uses DNA extracted from these exfoliated cells. BCA-1 has been used to examine more detailed CN data in bladder cancer patients than that provided by UroVysion, and shows some promise.^13, 14^ Unfortunately, obtaining sufficient cellular material for analysis is not always possible, hindering the clinical applicability of such tests. A small number of studies have therefore also investigated urinary cfDNA for UBC analysis with mixed results, and it has previously been suggested that due to its origin, cfDNA may be enriched for tumour-specific biomarkers with reduced contamination from germline DNA of non-cancerous cells.^15^

cfDNA in blood plasma, arising through cancer cell death (necrotic or apoptotic cells) and actively released DNA,^16, 17^ has been well studied as a liquid biopsy for various solid tumours. cfDNA in urine of bladder cancer patients has also been studied in this setting.^15^ This nucleic acid resource has been proposed to be predominantly necrotic in origin and quantitative changes in necrotic-specific cfDNA levels have been studied to discriminate between cancer and non-cancer patients.^18^

In this study, we report the utilisation of Affymetrix's OncoScan FFPE Assay Kit (Affymetrix, Santa Clara, CA, USA) for detailed genomic profiling of UBC using matched FFPE tumour-derived DNA, cellular DNA from urine cell pellets and cfDNA from urine supernatant. We demonstrate that the complex genomics and important clinically actionable aberrations that are evident in FFPE tumour material (currently the predominant diagnostic biospecimen for solid tumours) are echoed in urinary DNAs, and that the tumour genome is enriched in cfDNA compared with cellular DNA. These data illustrate that urinary cfDNA may represent a reliable resource for non-invasive genomic profiling of bladder cancer.

## Materials and methods

### Patients and biospecimens

FFPE tissue and urine samples were selected from the Bladder Cancer Prognosis Programme biospecimen repository on the basis of availability of matched FFPE, urine cell pellets and >5 ml urine supernatant for each patient (BCPP, ethics approval 06/MRE04/65).^19^ Patients were enrolled into BCPP on the basis of initial cystoscopic findings suggestive of primary UBC. All patients were newly diagnosed, had not received treatment for UBC before biospecimen collection and were subsequently treated according to contemporary guidelines. Inclusion and exclusion criteria are detailed elsewhere.^19^

Urine samples were obtained before transurethral resection of bladder tumour(s) (TURBT). Samples were placed on ice, centrifuged at 2000 r.p.m. for 10 min within 8 h of collection, and the supernatants and cell pellets were separated and stored at −80 °C. Representative FFPE samples were retrieved from local histopathology departments after clinical utilisation had ceased. Tumour grade and stage records were amended according to results of re-resection or cystectomy (where performed), and 10% of all FFPE samples collected underwent expert pathological review as part of routine quality assurance.

As patients were recruited to the BCPP cohort before a definitive diagnosis from TURBT, several patients were later found not to have UBC. Urinary supernatants from 12 of these patients were additionally included in this study as non-UBC controls and analysed alongside samples from the 23 confirmed UBC patients.

### DNA extraction

For the extraction of DNA from FFPE, seven sections were cut from blocks and mounted on Superfrost slides (Thermo Fisher Scientific, Waltham, MA, USA). The fourth section from each block was cut with a thickness of 3 *μ*m and stained with haematoxylin and eosin to identify tumour-enriched regions. This section was used as a template for macrodissection of the tumour-enriched region from the remaining unstained 6-*μ*m sections. DNA was extracted using the QIAamp DNA FFPE Tissue Kit (QIAGEN, Venlo, Netherlands), following the manufacturer's protocol.

Cellular DNA was extracted from urine pellets using the Urine DNA Isolation Kit for Exfoliated Cells or Bacteria (Norgen Biotek Corporation, ON, Canada) following the manufacturer's protocol. cfDNA was extracted from the supernatant of centrifuged urine using the Urine DNA Isolation Kit (Slurry Format; Norgen Biotek Corporation), following the manufacturer's protocol. Volumes available for extraction varied per patient, range of 6–27 ml (average of 18 ml).

DNAs from FFPE blocks, and cellular and cfDNA from urine were quantified using the Quant-iT PicoGreen dsDNA Assay Kit (Life Technologies, Carlsbad, CA, USA) following the manufacturer's protocol. The concentration of DNA stock was adjusted towards the recommended DNA input of 12 ng/*μ*l. Depending on the concentration of extracted DNAs, samples were diluted using reduced-EDTA TE buffer (10 mm Tris-HCl, 0.1 mm disodium EDTA, pH 8), concentrated using vacuum evaporation (where initial concentration was <12>2 ng/*μ*l) or concentrated by reprecipitation with sodium acetate and ethanol (where initial concentration was <2 ng/*μ*l).

### OncoScan assay

Up to 12 ng/*μ*l DNA was plated at 6.6 *μ*l per well (maximum of 79.2 ng DNA per well) into MicroAmp Optical 96-well reaction plates (Life Technologies), which were either used immediately or frozen at −20 °C until needed.

The OncoScan assay utilises molecular inversion probe (MIP) technology,^20^ for the identification of CN alterations, loss of heterozygosity (LOH) and recurrent clinically actionable somatic mutations (SMs). MIP probes in this assay enable the capture of the alleles of over 220 000 SNPs distributed across the whole genome, with increased probe density within ~900 cancer genes. They also enable detection of 74 frequently tested somatic mutations in *BRAF*, *KRAS*, *EGFR, IDH1, IDH2, PTEN, PIK3CA, NRAS* and *TP53*. The assay was undertaken following the recommended OncoScan protocol as previously described.^21^

OSCHP files were generated by the OncoScan Console software (Affymetrix), using data from fluorescence intensity (CEL) files generated during scanning of OncoScan chips. OSCHP files were used as inputs for the SM Viewer Software v1.01.16304 (Affymetrix) for the detection of SMs in each sample and Nexus Express for OncoScan 3.0.1 (BioDiscovery, Hawthorne CA, USA) for the analysis of CN aberrations and LOH.

### Data analysis

CN (loss, gain, biallelic loss or high amplification) and LOH calls were made in each sample independently, using the TuScan algorithm supplied with the OncoScan Console, followed by blinded manual interpretation of complex profiles. Grouped comparative analyses were then performed per patient to ensure consistent genome alignment for matched samples (some samples were reprocessed using BioDiscovery's FASST2 algorithm and traces realigned as required). These grouped analyses additionally allowed for confirmation of related genomics and a late stage sample identity check. SM calls were made separately using the SM Viewer software before genomic data were combined to give full genomic profiles. Somatic mutations identified in each patient were validated by next-generation sequencing technologies, subject to availability of material. Repeat calling of matched aberrations in corresponding patient samples allowed for further confirmation of findings. Before this study, a multi-site detailed platform validation study was completed. Results of this validation further inform the reliability of the OncoScan's genomic data.^21^

Following CN profile processing, CN aberrations that were present in matched cfDNA and cellular DNA samples were identified. The integer CN for each of these aberrations was estimated under the assumption of a normal diploid cell population mixed with a single homogeneous tumour clone. Aberrant cell fraction was then calculated on an aberration by aberration basis by formulas specific to the integer CN state of the aberration. This resulted in no fewer than three aberrations and their calculated aberrant cell fractions describing each matched set. To test whether the mean aberrant cell fraction of cfDNA was greater than that of cellular DNA, a one-sided paired *t*-test was performed, with the null hypothesis that there were no differences in the mean aberrant cell fraction between cfDNA and cellular DNA.

Genomic profiles were mined for the presence of genomic biomarkers listed in Van Allen's database of ‘tumour alterations relevant for genomics driven therapy' (TARGET: alterations that may have therapeutic, prognostic or diagnostic implications).^22^ Using the FFPE genomic profiles as the reference, the analytical sensitivities (for detection of FFPE-identified TARGET aberrations) were determined for each of the cfDNA and cellular DNA. The percentage sensitivities for each urinary DNA component were then compared using a two-sample *t*-test (where TARGET aberrations were identified in the matching FFPE sample and the quality of OncoScan data was sufficient). Samples were considered informative for the calculation of analytical sensitivities where TARGET aberrations were identified in matched FFPE samples and OncoScan data from the urinary DNA was of sufficient quality for accurate genomic analysis.

### Data availability

All microarray data (both raw CEL files and processed OSCHP files) have been publicly submitted to the ArrayExpress database (https://www.ebi.ac.uk/arrayexpress) under accession number E-MTAB-3841.^23^ Alongside the microarray data, pertinent meta-data has also been included detailing the tumour grading/staging and anonymised patient of sample origin.

### Orthogonal validation of somatic mutations

Where DNA availability allowed, SM calls made by the OncoScan assay were verified by PCR and next-generation sequencing. Sequencing was performed on a MiSeq (Illumina, San Diego, CA, USA) and data analysed by a custom pipeline built from cutadapt version 1.2.1;^24^ BWA-MEM version 0.7.12,^25^ SAMtools version 1.2^26^ and VarScan version 2.3.9.^27^

## Results

**Figure 1:**
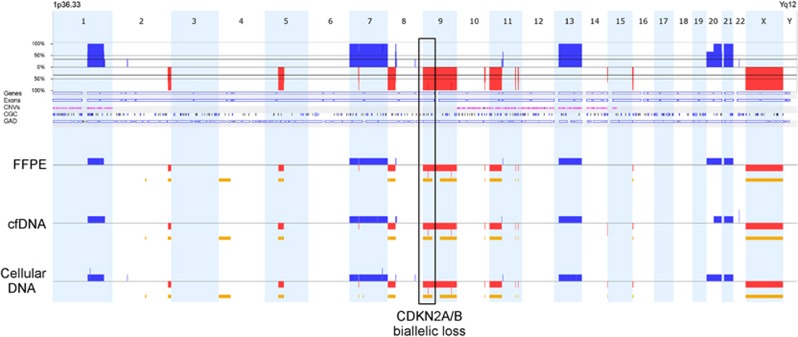
Example of genomic profiles from DNAs extracted from FFPE and cellular and cfDNA from the urine from patient 23 with a stage G2 pT1 NMIBC. Comparison of profiles shows consistent aberrations identified in all three samples (red=loss, blue=gain and yellow=LOH). Aberrations from all three sources show homozygous loss at 9p21.3 including CDKN2A/B; listed in the TARGET database. Biallelic inactivation of CDKN2A/B may predict response to CDK4/6 inhibitors for this patient.

Genomic profiles, where informative, show clear agreement of CN profiles between corresponding patient samples (Table 1; Figure 1). Tumour heterogeneity is evidenced by identification of disparate SMs and CN aberrations in matched samples (Table 1; patient 3), as well as by inconsistent aberrant levels for matched abnormalities in corresponding patient samples.

Somatic mutations identified by the OncoScan assay were validated using next-generation sequencing approaches as described above. Depending on DNA availability, mutations were confirmed either in remaining urine and FFPE DNAs or using DNA extracted from fresh-frozen tissue taken from the same patient tumours. This analysis allowed for confirmation of all mutations that were identified in all matched FFPE and urine DNA samples assayed. Only the *TP53* mutation in patient 3 (known to be clonal due to its absence in the initial FFPE DNA investigated) could not be identified in the matched fresh-frozen material and unfortunately there was insufficient FFPE or urine DNA available for this patient to allow further testing. However, as this mutation was identified independently in three separate DNAs from this patient using the OncoScan assay, we are confident that this is not an artefactual finding.

The tumour genome burden of the cfDNA and cellular DNA was calculated from the BAF of heterozygous SNPs and the predicted tumour genome burdens of urinary DNA samples were compared using a paired *t*-test (Materials and Methods). This data demonstrate a significantly greater tumour genome burden and lower germline DNA contamination in cfDNA over cellular DNA (*P*<0.001). These differences were also visualised in BAF plots, showing a lower detection rate of targetable genomic biomarkers in urinary cellular DNA. Furthermore, in six samples, cellular DNA was insufficient for elucidation of appropriate genomic information using the OncoScan FFPE assay kit (Table 1); in contrast, 22/23 urine supernatants studied provided sufficient cfDNA for accurate characterisation of genomic aberrations.

**Figure 2:**
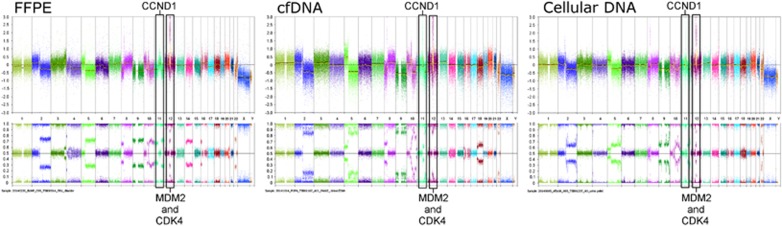
By examining the BAF plots of the SNP probes for patient 13, the highest aberrant cell load for this patient is observed in cfDNA (greatest separation in bottom plots). The number of TARGET aberrations (3; amplifications of CCND1 (may predict sensitivity to CDK4/6 inhibitors), MDM2 (may predict sensitivity to Nutlins and MDM2 inhibitors) and CDK4 (may predict sensitivity to CDK4/6 inhibitors)) is consistent across all sample types. A degree of tumour heterogeneity is also clear.

**Figure 3:**
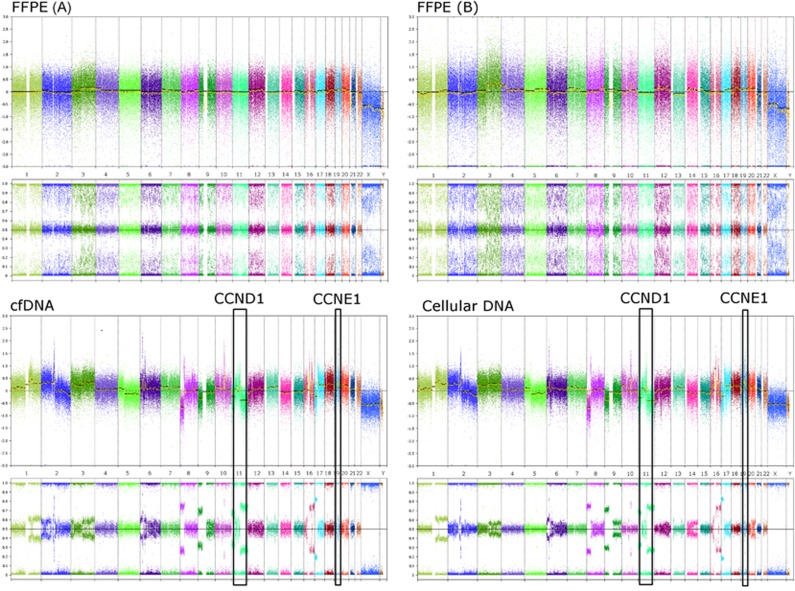
Patient 7. DNAs extracted from urine provide improved quality genomic data and clearer characterisation of the tumour profile than DNA from FFPE tumour material, despite repeat slides being cut and extracted. Two TARGET aberrations (CCND1 amplification (may predict sensitivity to CDK4/6 inhibitors) and CCNE1 amplification (may predict sensitivity to CDK2 inhibitors)) were observed in both cfDNA and urinary cellular DNA however none of these aberrations were independently called in two separate DNAs from FFPE tumour material.

In several samples, cfDNA from urine supernatant was observed as having a greater aberrant tumour genome load than FFPE material (Figures 2 and 3). For patient 6 (Table 1), there was insufficient DNA from FFPE tumour material to yield a result; only the cfDNA sample from this patient evidenced the cancer genome.

Analytical sensitivities for detection of key FFPE-identified genomic biomarkers were calculated, as described above. These data show cfDNA to have an average analytical sensitivity of ~90% (range 0–100%, 17 informative samples) for detection of FFPE-identified aberrations. Cellular DNA from the urine has an average analytical sensitivity of 61% (range 0–100%, 15 informative samples). These data demonstrate a significantly greater analytical sensitivity for cfDNA over cellular DNA from urine (*P*<0.04; Table 1).

Both the FFPE samples and cfDNA from urine, which were examined in our data set, showed an average of 2.3 TARGET aberrations per patient (range 0–12, 22 samples informative). DNA from urine cell pellets showed an average of 1.3 TARGET aberrations per patient (range 0–4, 18 samples informative; Table 1).

**Figure 4:**
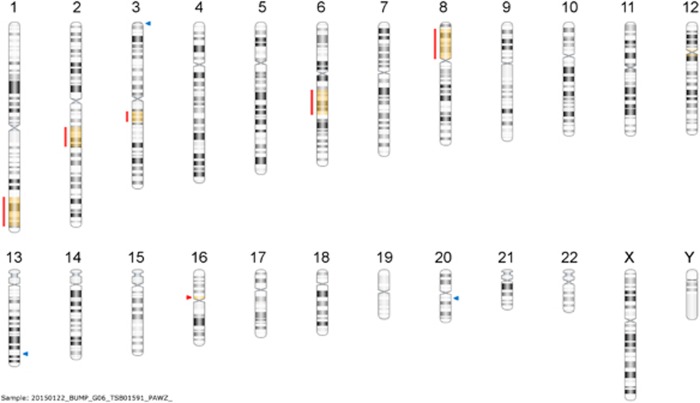
Genomic profile from urinary cfDNA of non-UBC patient with prostatic duct carcinoma confirms that detection of a tumour genome in urinary DNA is not bladder cancer specific. Red lines highlight regions of CN loss with resulting loss of heterozygosity indicated by yellow colouring of the chromosomal regions. Blue and red arrows indicate sub-microscopic germline CN gains and losses, respectively.

Following blinded analysis, 11 out of 12 urinary cfDNAs from non-UBC patients showed genomics consistent with the germline genome only. One patient, later revealed to harbour prostatic duct carcinoma, showed CN alterations consistent with malignancy (Figure 4).

## Discussion

Recent developments in the genomic profiling of tumours have led to rapid growth in our understanding of the genetic basis of cancer.^28^ Genomic biomarkers are increasingly being identified as indicators for disease prognosis or diagnosis, or for predicting response to targeted therapies.^22^ Non-invasive identification of these biomarkers is an area of intense interest and shows potential to significantly improve patient care. Many groups have looked at cell-free tumour DNA circulating in the blood or other body fluids as important liquid biopsy resources for non-invasive tumour profiling^9, 15^ and, recurrently, specific genomic biomarkers for UBC diagnosis have been identified in cells exfoliated into the urine.

Here we demonstrate the clinical utility of urinary DNA from UBC patients and support the hypothesis that cfDNA from urine provides an improved resource for non-invasive genomic profiling of UBC when compared with urine cellular DNA. A previous study looking more simply at microsatellite analyses in urinary DNAs from 44 UBC patients suggested a higher analytical sensitivity when using cfDNA over DNA from urinary cell pellets.^15^ Here we have elaborated on these findings and illustrated the use of cfDNA to capture genomic complexity across the UBC tumour genome using a comprehensive genomic profiling platform well suited to small quantities of highly degraded DNA. Our data show that cfDNA from UBC patients is highly representative of the tumour genome and has a consistently higher tumour burden than DNA from exfoliated whole cells (*P*<0.001), allowing greater detection of important tumour-specific biomarkers (*P*<0.04). It is hypothesised that this increased representation of the tumour genome in the cfDNA is a result of the increased rate of necrosis for tumour cells relative to normal urothelium.

Our data show evidence of tumour heterogeneity, illustrated by variations in genomics between matched patient samples (all taken at diagnosis). Our data also demonstrate that biomarkers present in FFPE material can be accurately and robustly identified in urinary cfDNA with a higher analytical sensitivity (90%) than urine cellular DNA (61%), *P*<0.04.

Analyses of cfDNAs from control patients with UBC symptoms show CN aberrations only in the presence of other malignancies. These data suggest high specificity of urinary cfDNA for the detection of malignancy, but confirms that genomic analysis of cfDNA is not UBC specific. Previous authors have investigated nucleic-acid-based biomarkers in the body fluids of patients with a variety of urologic malignancies, recently reviewed by Ralla *et al.*^9^

Genomic CN aberrations in exfoliated cells from urine have recently been shown to pre-date cancer development by ~3 years in some patients;^29^ it is possible that cfDNA would not mirror these predictive CN aberrations in the absence of necrotic tumour material. The investigation of such phenomena was beyond the remit of this study, but is the subject of ongoing analyses.

The data presented herein represent a proof of principle that urinary cfDNA from UBC patients represents a promising resource for the identification of complex cancer genomes and specific targetable aberrations when profiled using the OncoScan FFPE assay kit. Urinary cfDNA may thus have utility for non-invasive disease diagnosis, surveillance, prognostication and prediction or monitoring of treatment responses.

## Conclusions

cfDNA from urine supernatant allows for accurate and detailed whole-genome profiling of UBCs. It has a higher tumour genome burden than urine cellular DNA, and shows a higher analytical sensitivity for detection of important genomic biomarkers present in the tumour genome. cfDNA from the urine thus provides a promising resource for non-invasive genomic profiling of UBCs to help guide patient management without invasive sampling, thereby potentially improving patient care.

## Figures and Tables

**Table 1:** Tumour staging data for 23 patients with UBC together with information on TARGET aberrations identified

*Patient number*	*Tumour stage*	*Grade*	*DNA source*	*Number of TARGET aberrations identified*	*Sensitivity for TARGET mutations present in FFPE (%)*	*Details of TARGET aberrations*	*Comments*
1	pT2+	G3	FFPE tumour sections	2	NA	KIT amp; CDKN2A/B biallelic loss	
			Urine supernatant	2	100	KIT amp; CDKN2A/B biallelic loss	
			Urine cell pellet	NA	NA	NA	DNA insufficient for OncoScan
2	pT2+	G3	FFPE tumour sections	5	NA	AKT2 amp; CDK4 amp; MCL1 amp; MDM2 amp; RAF1 amp	
			Urine supernatant	5	100	AKT2 amp; CDK4 amp; MCL1 amp; MDM2 amp; RAF1 amp	
			Urine cell pellet	0	0	NA	
3	pT2+	G3	FFPE tumour sections	3	NA	PIK3CA:c.1633G>A (p.(E545K)); PIK3CA:c.1624G>A (p.(E542K)); CDKN2A/B biallelic loss	Differences in somatic mutations identified in urine and FFPE samples highlights tumour heterogeneity. Mutations in urine cfDNA consistent with FFPE sections from deeper tumour material (data not shown)
			Urine supernatant	3	67	PIK3CA:c.1633G>A (p.(E545K)); TP53:c.524G>A (p.(R175H)); CDKN2A/B biallelic loss	
			Urine cell pellet	3	67	PIK3CA:c.1633G>A (p.(E545K)); TP53:p.R175H; TP53:c.524G>A (p.(R175H)) CDKN2A/B biallelic loss	
4	pT2+	G3	FFPE tumour sections	0	NA		Clear and consistent CN aberrations evident for both cfDNA and FFPE DNA
			Urine supernatant	0	NA		
			Urine cell pellet	0	NA		
5	pT2+	G3	FFPE tumour sections	12	NA	AKT2 amp; AURKA amp; BRAF amp; CCND3 amp; CCNE1 amp; CDK6 amp; CRKL amp; EGFR amp; FGFR1 amp; MAPK1 amp; MCL1 amp; MET amp	
			Urine supernatant	12	100	AKT2 amp; AURKA amp; BRAF amp; CCND3 amp; CCNE1 amp; CDK6 amp; CRKL amp; EGFR amp; FGFR1 amp; MAPK1 amp; MCL1 amp; MET amp	
			Urine cell pellet	0	0		Apparently representative of germline genome only
6	pT2+	G3	FFPE tumour sections	NA	NA		DNA insufficient for OncoScan
			Urine supernatant	1	100	MCL1 amp	In absence of FFPE results, assume cfDNA to be representative of tumour
			Urine cell pellet	0	0		Apparently representative of germline genome only
7	pT2+	G3	FFPE tumour sections	0	NA	Quality insufficient to call aberrations	
			Urine supernatant	2	100	CCND1 amp; CCNE1 amp	In absence of FFPE results, assume urine DNA aberrations to be representative of tumour
			Urine cell pellet	2	100	CCND1 amp; CCNE1 amp	In absence of FFPE results, assume urine DNA aberrations to be representative of tumour
8	pT2+	G3	FFPE tumour sections	3	NA	FGFR1 amp; MYC amp; PIK3CA:c.3140A>G (p.(H1047R))	
			Urine supernatant	3	100	FGFR1 amp; MYC amp; PIK3CA:c.3140A>G (p.(H1047R))	
			Urine cell pellet	1	33	PIK3CA:c.3140A>G (p.(H1047R))	
9	pTa	G1	FFPE tumour sections	0	NA		
			Urine supernatant	0	NA		
			Urine cell pellet	0	NA		
10	pT2+	G3	FFPE tumour sections	0	NA		
			Urine supernatant	0	NA		
			Urine cell pellet	NA	NA		DNA insufficient for OncoScan
11	pTa	G2	FFPE tumour sections	1	NA	CDKN2A biallelic loss	
			Urine supernatant	NA	NA		Quality of OncoScan data too poor for accurate analysis
			Urine cell pellet	NA	NA		DNA insufficient for OncoScan
12	pT2+	G3	FFPE tumour sections	1	NA	TP53:c.844C>T (p.(R282W))	
			Urine supernatant	1	100	TP53:c.844C>T (p.(R282W))	
			Urine cell pellet	1	100	TP53:c.844C>T (p.(R282W))	
13	pT2+	G3	FFPE tumour sections	3	NA	CCND1 amp; CDK4 amp; MDM2 amp	
			Urine supernatant	3	100	CCND1 amp; CDK4 amp; MDM2 amp	
			Urine cell pellet	3	100	CCND1 amp; CDK4 amp; MDM2 amp	
14	pTa	G1	FFPE tumour sections	1	NA	PIK3CA:c.3140A>G (p.(H1047R))	
			Urine supernatant	1	100	PIK3CA:c.3140A>G (p.(H1047R))	
			Urine cell pellet	1	100	PIK3CA:c.3140A>G (p.(H1047R))	
15	pT2+	G3	FFPE tumour sections	1	NA	EGFR amp	
			Urine supernatant	0	0		
			Urine cell pellet	0	0		
16	pT1	G3	FFPE tumour sections	8	NA	BRAF amp; CCND3 amp; CDK6 amp; EGFR amp; MAPK3 amp; MET amp; MYC amp; RAF1 amp	
			Urine supernatant	8	100	BRAF amp; CCND3 amp; CDK6 amp; EGFR amp; MAPK3 amp; MET amp; MYC amp; RAF1 amp	
			Urine cell pellet	4	50	CCND3 amp; MAPK3 amp; MYC amp; RAF1 amp	
17	pT1	G2	FFPE tumour sections	1	NA	CCND1 amp	
			Urine supernatant	1	100	CCND1 amp	
			Urine cell pellet	1	100	CCND1 amp	
18	pT2+	G3	FFPE tumour sections	4	NA	CCNE1 amp; RAF1 amp; CDKN2A/B biallelic loss; PIK3CA:c.1624G>A (p.(E542K))	
			Urine supernatant	4	100	CCNE1 amp; RAF1 amp; CDKN2A/B biallelic loss; PIK3CA:c.1624G>A (p.(E542K))	
			Urine cell pellet	4	100	CCNE1 amp; RAF1 amp; CDKN2A/B biallelic loss; PIK3CA:c.1624G>A (p.(E542K))	
19	pT2+	G2	FFPE tumour sections	3	NA	CCND1 amp; CDKN2A/B biallelic loss; TSC1 biallelic loss	
			Urine supernatant	2	67	CCND1 amp; CDKN2A/B biallelic loss	
			Urine cell pellet	2	67	CCND1 amp; CDKN2A/B biallelic loss	
20	pTa	G3	FFPE tumour sections	0	NA		
			Urine supernatant	0	NA		
			Urine cell pellet	NA	NA		DNA insufficient for OncoScan
21	pTa	G1	FFPE tumour sections	0	NA		
			Urine supernatant	0	NA		
			Urine cell pellet	0	NA		
22	pTa	G3	FFPE tumour sections	1	NA	CDKN2A/B biallelic loss	
			Urine supernatant	1	100	CDKN2A/B biallelic loss	
			Urine cell pellet	NA	NA		DNA insufficient for OncoScan
23	pT1	G2	FFPE tumour sections	1	NA	CDKN2A/B biallelic loss	
			Urine supernatant	1	100	CDKN2A/B biallelic loss	
			Urine cell pellet	1	100	CDKN2A/B biallelic loss	

Abbreviations: FFPE, formalin-fixed paraffin embedded; NA, not applicable.

Analytical sensitivity (for detection of FFPE-identified aberrations) is indicated. CN probes are mapped to Genome Reference Consortium human genome build 37 (GRCh37). Reference sequences used for somatic mutations listed are TP53 (NM_000546.5) and PIK3CA (NM_006218.2).
